# Epidemiological and clinical characteristics of children hospitalized due to influenza A and B in the south of Europe, 2010–2016

**DOI:** 10.1038/s41598-019-49273-z

**Published:** 2019-09-06

**Authors:** Mireia Jané, Maria José Vidal, Nuria Soldevila, Arancha Romero, Ana Martínez, Nuria Torner, Pere Godoy, Cristian Launes, Cristina Rius, Maria Angeles Marcos, Angela Dominguez

**Affiliations:** 1 Public Health Agency of Catalonia. Department of Health, Barcelona, Spain; 2 CIBER Epidemiology and Public Health (CIBERESP), Carlos III Institute of Health, Madrid, Spain; 3 Department of Medicine, University of Barcelona, Barcelona, Spain; 4 Sant Joan de Deu Children’s Hospital, Esplugues de Llobregat, Spain; 5 Public Health Agency of Barcelona, Barcelona, Spain; 6 Hospital Clínic of Barcelona, Barcelona, Spain

**Keywords:** Influenza virus, Paediatrics

## Abstract

Influenza produces annual epidemics that affect 5–15% of the world population. Complications and hospitalizations are more frequent in childhood. This study describes and analyses the epidemiological and clinical characteristics of children hospitalized due to confirmed influenza in influenza surveillance sentinel hospitals in Catalonia. Retrospective descriptive study conducted in six influenza seasons (2010–2011 to 2015–2016) in persons aged 0–17 years diagnosed with laboratory-confirmed influenza requiring hospitalization. 291 cases were notified to the health authorities: 79.4% were due to the influenza A virus and 20.6% to the B virus. The most common subtype was H1N1 with 57.6% of cases: 52.6% were male, 56.7% were aged <2 years, and 24.4% were aged <1 year. 62.2% of cases had pneumonia, 26.8% acute respiratory distress syndrome and 11.7% bacterial pneumonia. 5.8% of cases were vaccinated and 21.3% required intensive care unit admission, of whom 54.8% were aged <2 years. There were 3 deaths, all with influenza A infection. Influenza A cases were younger than influenza B cases (OR 3.22; 95% CI: 1.73–6.00). Conclusion: Children aged <2 years are especially vulnerable to the A H1N1 virus, including those without pre-existing chronic disease. These results are relevant for the planning of vaccination programs to improve maternal and child health.

## Introduction

Influenza, an acute viral infectious disease that primarily affects the upper respiratory tract, is caused by the influenza virus, which belongs to the *Orthomyxoviridae* family. There are three types of influenza viruses, which commonly infect humans: A, B and C. The first two are responsible for annual seasonal epidemics. Type A influenza viruses are divided into subtypes depending on the composition of hemagglutinin (H) and neuraminidase (N), two proteins on the virus surface. There are several subtypes of influenza A but, currently, the viruses that circulate and affect humans are subtypes A (H3N2) and A (H1N1). Subtype A (H1N1) pdm09 was first identified in 2009, in the first influenza pandemic of the 21st century, and since then has circulated along with other seasonal viruses in the post-pandemic period^1^. Influenza produces annual epidemics that affect between 5 and 15% of the world population. It is estimated that between 3 and 5 million people have serious influenza with complications and hospital admission annually and approximately 250,000–500,000 people die annually due to influenza^2^. Influenza symptoms vary widely from mild to lower respiratory tract infection such as bronchitis or complicated pneumonia, leading to respiratory and multiorgan failure and death. Influenza may affect people of any age, but complications and hospitalizations are more frequent in childhood, especially in children aged <5 years, people with chronic disease and people aged ≥65 years^1,3^. Studies have shown that children and people with chronic underlying disease are more susceptible to bacterial superinfection secondary to influenza^1,4^. Some reports suggest that children may more easily introduce and transmit influenza in the community^1,5,6^.

A surveillance program of influenza and other acute respiratory infections (PIDIRAC) was initiated in Catalonia in the 1999–2000 season, based on a network of sentinel primary care physicians (general practitioners and paediatricians) who report influenza-like activity detected in their reference population daily. The aim is to study the behaviour of influenza, detect the beginning of epidemics and characterize the circulating viruses in a representative sample of the population^7^. Since the 2005–2006 season, the PIDIRAC has formed part of the Spanish influenza surveillance sentinel system, coordinated by the National Center for Epidemiology and the National Center for Microbiology, both of the Carlos III Health Institute^8^ and which, since 2008 is part of the European Influenza Surveillance Network (EISN) coordinated by the European Center for Disease Control (ECDC) which is responsible for influenza surveillance in Europe. As a result of the 2009 pandemic, in the 2010–2011 season, the surveillance of severe hospitalized cases of confirmed influenza was introduced, using a sentinel hospital system that at that time included 12 hospitals geographically distributed throughout all Catalan health care regions. In Catalonia influenza vaccination is not recommended in childhood except if they have chronic disease that increases the risk of complications. The aim is to determine the activity of influenza viruses and the severity of hospitalized cases^9^. Various studies have analysed the distribution of influenza in the general population, vaccine effectiveness and hygienic preventive measures in our setting, but the characteristics and distribution of severe disease in children are not known^10–13^. The main objective of this study was to describe and analyse the epidemiological and clinical characteristics of children hospitalized due to confirmed influenza A or B virus infection in the sentinel hospitals in the six post-pandemic seasons in Catalonia.

## Methods

We carried out a retrospective descriptive study in six influenza seasons (2010–2011 to 2015–2016) in children and adolescents aged 0–17 years hospitalized due to severe acute influenza virus infection.

The surveillance system of severe hospitalized influenza in Catalonia, a region in the northeast of Spain with 7.5 million inhabitants, included 12 hospitals covering a total population of 4,644,543 during the study period (62% of the Catalan population)^9,14^.

Following a standardised regional protocol with a formal case definition, the clinician made the decision on who was a case. A hospitalized case of severe influenza was defined as a case of laboratory-confirmed influenza virus infection that required hospitalization due to pneumonia, acute respiratory distress syndrome, septic shock, multiorgan failure or any other serious condition, including intensive care unit (ICU) admission or death or who developed these criteria during hospitalization for another reason.

Laboratory confirmation was made using a sample obtained by aspirate or nasopharyngeal swab that was subject to PCR and/or culture. All hospitalized cases were followed to determine the disease progression.

The variables studied were collected from each reported case using a Catalan epidemiological surveillance network structured questionnaire. The primary source of information was the medical record. The variables studied were: sex, age, pre-existing chronic disease (asthma, chronic respiratory disease, obesity, diabetes, kidney disease, immunodeficiency, cardiovascular disease, liver disease), date of symptom onset and date of hospital admission, ICU admission, complications (pneumonia, acute respiratory distress syndrome, multiorgan failure and septic shock), antiviral treatment received, seasonal influenza vaccination status and viral type/subtype (A H1N1 pdm09, A H3N2, A without subtyping and B).

### Statistical analysis

A bivariate analysis was performed to identify factors associated with the type of influenza virus (A or B). Associations between clinical and epidemiological characteristics and influenza type were determined by calculating the odds ratio (OR) and 95% confidence intervals (CI) adjusting by the population of each season. The differences between influenza A subtypes and influenza B were assessed using the Chi-square test. The statistical analysis was made using the SPSS version 24.0 statistical package and the R v3.5.0 statistical software.

### Ethical considerations

All data used in the analysis were collected during routine public health surveillance activities, as part of the legislated mandate of the Health Department of Catalonia, the competent authority for the surveillance of communicable diseases, which is officially authorized to receive, treat and temporarily store personal data on cases of infectious disease. Therefore, all study activities formed part of public surveillance and were thus exempt from institutional board review and did not require informed consent. All data were fully anonymized.

### Ethical approval

This article does not contain any studies with human participants or animals performed by any of the authors.

## Results

During the six seasonal influenza seasons between 2010–2011 and 2015–2016, 291 confirmed cases of influenza requiring hospitalization were reported to the General Subdirectorate of Surveillance and Response to Public Health Emergencies, with ages ranging from 0 to 17 years.

**Figure 1:**
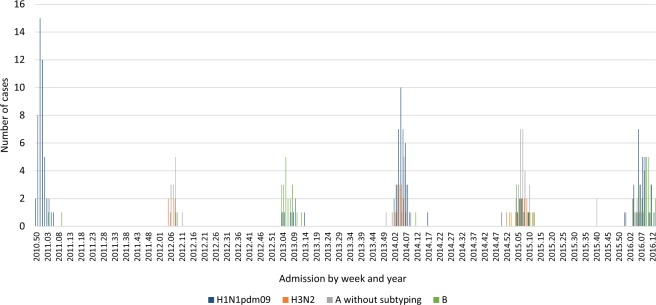
Weekly hospitalizations due to influenza in children aged <18 years according to season and week of admission. Catalonia, 2010–2011 to 2015–2016.

Figure 1 shows the weekly cases hospitalized in the six seasons. There was a predominance of influenza A(H1N1)pdm09 subtype in cases hospitalized in the 2010–2011 and 2013–2014 seasons, with hospitalizations reaching a peak in December, January and February. In seasons with a predominance of influenza B viruses (season 2012–13 and 2015–16), there was co-circulation with influenza A(H1N1) pdm09 subtype.

Of the 291 hospitalized cases, 79% tested positive for influenza A virus and 21% for influenza B. The most frequent subtypes were H1N1 pdm09 (57.8%) and H3N2 (13.1%), while 29.1% of cases could not be subtyped. Of the total, 52.6% were male, 56.7% were aged <2 years, 24.4% were aged <1 year and the mean age was 3.3 years (±SD 4), 16.8% had chronic disease, including asthma (28.6%), cardiovascular disease (26.5%), immunosuppressive disease (20.4%) and chronic respiratory disease (14.3%). The mean length of hospital stay was 9.2 days (±SD13.8). The mean time between symptom onset and hospital admission was 3.5 days (±SD 2.9), and 70% received antiviral treatment with oseltamivir or zanamivir, of whom 28.7% received it within 48 hours after symptom onset. The mean time between symptom onset and initiation of antiviral treatment was 5.2 days (±SD 4.2). The most frequent complications were pneumonia (62.2%), acute respiratory distress syndrome (26.8%) and bacterial pneumonia (11.7%).

ICU admission was required in 21.3% of cases (21.7% of influenza A cases and 19.7% of influenza B cases), with a mean length of stay of 12.8 days (±SD 9.7): 54.8% of those admitted to the ICU were aged <2 years.

The vaccination rate was 5.8% in all children and 27.1% in cases with chronic disease.

The clinical and epidemiological differences of cases hospitalized due to influenza type A and B are shown in Table 1. Cases of influenza A were significantly younger (OR 3.22; 95% CI: 1.73–6.00): 62.6% of cases of influenza A were aged <2 years, while 63.9% of cases of influenza B were aged 3–17 years. No differences were found according to sex (OR 1.05; 95% CI: 0.57–1.92). Differences in the mean hospital stay were not significant: influenza A 9.89 days (±SD18.37) vs. influenza B 8.49 days (±SD9.08). There were no significant differences in the mean time from symptom onset to hospital admission according to virus type.

**Table 1:** Comparison of clinical and epidemiological characteristics of children aged <18 years hospitalized due to influenza type A or B virus. Catalonia, seasons 2010–2011 to 2015–2016.

	Influenza A	Influenza B	OR (95% CI)
	(n = 230)	(n = 61)	
	N (%)	N (%)	
Female	111 (48.3)	27 (44.3)	1.05 (0.57–1.92)
Male	119 (51.7)	34 (55.7)	Ref.
Age			
0–2 years	144 (62.6)	22 (36.1)	3.22 (1.73–6.00)
3–17 years	86 (37.4)	39 (63.9)	Ref.
Mean age ± SD *	3.1 ± 4.0	4.4 ± 3.9	0.92 (0.86–0.99)
Pre-existing chronic disease	34 (14.8)	15 (24.6)	0.53 (0.26–1.05)
COPD	4 (1.7)	3 (4.9)	0.35 (0.08–1.60)
Asthma Ω	10/105 (9.5)	4/42 (9.5)	0.99 (0.29–3.37)
Obesity	3 (1.3)	1 (1.6)	0.73 (0.07–7.18)
Diabetes	1 (0.4)	1 (1.6)	0.27 (0.02–4.49)
Chronic renal failure	2 (0.9)	1 (1.6)	0.51 (0.05–5.78)
Chronic liver failure	1 (0.4)	0 (0)	—
Immunosuppression	9 (3.9)	1 (1.6)	2.70 (0.33–22.17)
Cardiovascular disease	8 (3.5)	5 (8.2)	0.38 (0.12–1.21)
Other risk factors Ω	1/26 (3.8)	2/10 (20.0)	0.17 (0.01–2.03)
ICU admission	50 (21.7)	12 (19.7)	1.09 (0.54–2.22)
Hospital stay (days) mean ± SD	9.9 ± 18.4	8.40 ± 9.0	1.01 (0.98–1.03)
Pneumonia	152 (67.0)	41 (68.3)	0.93 (0.51–1.72)
Acute respiratory distress syndrome	59 (26.6)	19 (32.2)	0.77 (0.41–1.43)
Antiviral treatment ≤48 h	45 (20.3)	11 (18.3)	1.47 (0.65–3.32)
>48 h	113 (50.9)	26 (43.3)	1.63 (0.86–3.10)
No	64 (28.8)	23 (38.3)	Ref.
Mean days from symptom onset to treatment	4.9 ± 3.4	5.5 ± 5.0	1.02 (0.92–1.12)
Vaccination	13 (5.7)	4 (6.9)	0.83 (0.26–2.66)

*Age as a continuous variable is shown only as a descriptive variable.

Ω: In clinical conditions, asthma and other risk factors, the denominator data is written to show the total of completed questionnaires.

Table 2 shows cases requiring ICU admission: 80.6% had influenza A virus infection (60% due to subtype A (H1N1) pdm09) and 19.4% had influenza B virus infection. In children with pre-existing chronic disease, 16% had influenza A infection and 33.3% influenza B: 30.6% were aged <1 year.

**Table 2:** Characteristics of children aged <18 years with ICU admission according to type and subtype of influenza virus. Catalonia, seasons 2010–2011 to 2015–2016.

	Influenza A			Influenza B	P value
	H1N1pdm09 (n = 30)	H3N2 (n = 7)	A without subtyping (n = 13)	(n = 12)	
	N (%)	N (%)	N (%)	N (%)	
Age					0.37
<1 year	9 (30.0)	4 (57,1)	2 (15.4)	4 (33.3)	
1–2 years	6 (20.0)	3 (42.9)	5 (38.5)	1 (8.3)	
3–5 years	8 (26.7)	—	2 (15.4)	4 (33.3)	
6–11 years	4 (13.3)	—	3 (23.1)	3 (25.0)	
12–17 years	3 (10.0)	—	1 (7.7)	—	
Mean age ± SD	3.9 ± 4.6	0.6 ± 0.8	3.8 ± 4.0	3.3 ± 3.2	
Pre-existing chronic disease (agglutinated for ICU cases)	3 (10.0)	1 (14.3)	4 (30.8)	4 (33.3)	0.22
Mean ICU stay (days)	12.3 ± 9.7	8.3 ± 2.4	15.4 ± 11.5	15.1 ± 15.6	0.33
Pneumonia	19 (65.5)	2 (28.6)	10 (76.9)	7 (58.3)	0.19
Bacterial coinfection	6 (37.5)	—	4 (57.1)	1 (20.0)	0.39
Multiorgan failure	3 (11.5)	—	1 (7.7)	—	0.54
Acute respiratory distress syndrome	13 (50.0)	5 (71.4)	4 (30.8)	4 (36.4)	0.31
Death	2 (6,7)	—	1 (7.7)	—	

Of the 62 children admitted to the ICU, 61.3%, 41.9%, 17.7% and 6.4% had pneumonia, acute respiratory distress syndrome, bacterial coinfection or multiorgan failure, respectively: 81.6% of cases of pneumonia, 90.9% of bacterial pneumonia and 84.6% of acute respiratory distress syndrome occurred in influenza A cases. In more than half of all influenza A cases, the cause was the H1N1 subtype. All cases presenting multiorgan failure had influenza A and, of these, 75% were due to subtype A (H1N1) pdm09. The mean ICU stay for influenza A (H1N1) pdm09 and B were longer (12.3 days (±SD 9.7) and 15.1 days (±SD 15.6), respectively) than for influenza A (H3N2) (8.3 days ± SD 2.4), although the differences were not significant.

There were three deaths (<1 year, 1 year and 8 years, respectively), all infected by influenza type A (two H1N1 pdm09 and one A without subtyping). The time between symptom onset and hospitalization admission was 7, 3 and 13 days, respectively. None had chronic disease. Two received antiviral treatment, one within 48 hours after symptom onset. One death occurred without presenting complications; another occurred after presenting pneumonia with acute respiratory distress syndrome, bacterial pneumonia and multiorgan failure; the third death presented bacterial pneumonia. None had received the influenza vaccine.

## Discussion

Our results show the clinical and epidemiological characteristics of persons aged 0–17 years hospitalized with severe conditions due to influenza A and B in the post-pandemic seasons between 2010–2011 and 2015–2016 in Catalonia. Four-fifths of cases were infected by influenza A and 20% by influenza B.

Although not significant, hospitalization due to influenza was more frequent in males than in females, as reported by other studies^1,15–17^. There are few studies and no firm conclusions can be drawn, but there are suggestions that differences in sex and morbidity and mortality due to influenza may be related to endocrine-immunological interactions and behavioural and genetic aspects^18,19^.

More than half the children hospitalized were aged <2 years, in line with other studies that found a higher risk of hospitalization in younger children^1,15,20^. Age was also a significant differential factor when we analysed virus subtypes. The influenza A (H1N1) pdm09 subtype was more frequent and aggressive in children aged <2 years (63.1%), while influenza B was more frequent in children aged 3–11 years (58.3%).

One in five children had chronic disease. Although not significant, 24.6% of cases of influenza B had pre-existing chronic disease compared with 14.8% of cases of influenza A. This is in line with other studies, such as that by Mancinelli *et al*.^1^ who found that children with cancer were more susceptible to influenza B, and the study by Acar *et al*.^21^ which found influenza B infection is more frequent in children with pulmonary, cardiac or immune system disorders.

Around 20% of children required ICU admission. Of these, more than three-quarters were infected with the influenza A virus, with the H1N1 pdm09 subtype being found in more than half the cases. The higher frequency of type A and, specifically, the H1N1 subtype, among cases admitted to the ICU, is in line with other published studies^1,21–23^. This suggests disease severity is associated with influenza A infection, as in our study, in which the three deaths were caused by influenza A, and two by subtype H1N1 pdm09.

The percentage of cases requiring ICU admission were similar in influenza A and influenza B cases (21.7% and 19.7%, respectively). The study by Tran *et al*.^24^ obtained similar percentages between influenza A and influenza B cases (12.7% and 12.6%, respectively); however, these percentages were lower than those obtained in the present study but, in the study by Tran *et al*. all hospitalized children were included and in our study severe hospitalized children were included.

Pneumonia and acute respiratory distress syndrome were the most frequent complications, as observed in other studies^6,25^. A small percentage of hospitalized cases were vaccinated against seasonal influenza and only one in four children with pre-existing chronic disease were vaccinated. Jules *et al*. described a vaccination coverage of <50%^26^, even though influenza vaccination is included in the routine vaccination schedule: before the vaccine was included in the schedule, the vaccination coverage was <6% in children.

Children have a higher incidence of influenza and a higher hospitalization rate than adults^27^ and are an important reservoir in its transmission^6,28^, generating a high socio-economic impact due to school and work absenteeism, and to greater pharmaceutical expenditure and use of health resources^6^. For these reasons, recommendations on the inclusion of routine childhood vaccines in vaccination programs, especially in young children, are gaining weight. European countries such as Austria, Finland, Latvia and Slovakia have introduced routine influenza vaccination for all children from 6–7 months, while in the United Kingdom it is administered systematically from 2 years of age and before this in children at risk of influenza^29–31^. The United States, Canada and Japan have also included influenza vaccination from 6 months of age^32–34^. Sugaya *et al*. evaluated the inclusion of influenza vaccination in the 2013–2016 seasons in children aged 6 months-15 years in Japan and found a vaccine effectiveness (VE) in disease prevention of 51% for influenza A and 32% for influenza B; the VE in preventing hospitalizations was 52% for influenza A and 28% for influenza B^35^.

Currently, vaccination of people aged <60 years with a high risk of complications from influenza is recommended in Catalonia^36^, but this does not include children without chronic disease, especially those aged <2 years, who are more vulnerable. It is important to follow up on these data in the future and analyze this aspect in more depth. Influenza vaccination as a preventive measure for all children from 6 months of age and, especially, for the youngest children, is a key measure that may benefit individuals and the whole population and has been shown to be a strategy with a good cost-effectiveness ratio^27,30,37^. Currently, there seem to be no studies supporting the administration of influenza vaccine or antiviral treatment in children aged <6 months, due to poor immunogenicity. Therefore, in these cases, to avoid maternal disease, women should be vaccinated during pregnancy, since this may transmit antibodies that protect newborns during the first months of life. Thus, it is essential to increase vaccination coverages in this group^38^.

This study, like all descriptive studies, had strengths and limitations. The results provide a detailed summary of characteristics of children hospitalized with severe infection. The main limitation is that it is a retrospective study and it was not possible to collect more specific information about the clinical cases, such as fever, cough, wheezing, runny nose, odynophagia, malaise, vomiting and diarrhoea.

In conclusion, this is the first study to describe the clinical and epidemiological characteristics of children hospitalized due to influenza in Catalonia, and the differences observed according to the viral types and subtypes. The results show that children aged <2 years are especially vulnerable to the A H1N1pdm09 virus, even though they have no pre-existing chronic disease. These results should be taken into account in vaccination programs and in planning for and making improvements in maternal and child health.

## Data Availability

All data generated or analyzed during the study are included in this manuscript.
